# Increased topographical variability of task-related activation in perceptive and motor associative regions in adult autistics

**DOI:** 10.1016/j.nicl.2014.02.008

**Published:** 2014-02-25

**Authors:** Marie-Pier Poulin-Lord, Elise B. Barbeau, Isabelle Soulières, Oury Monchi, Julien Doyon, Habib Benali, Laurent Mottron

**Affiliations:** a Centre d'Excellence en Troubles Envahissants du Dèc)veloppement de l'Universitèc) de Montrèc)al (CETEDUM), Montrèc)al, QC, Canada; b Centre de recherche de l'Institut universitaire en santèc) mentale de Montrèc)al, Montrèc)al, QC, Canada; c Dèc)partement de Psychiatrie, Universitèc) de Montrèc)al, Montrèc)al, QC, Canada; d Dèc)partement de Psychologie, Universitèc) du Quèc)bec à Montrèc)al, QC, Canada; e Centre de Recherche, Institut Universitaire de Gèc)riatrie de Montrèc)al, Montrèc)al, QC, Canada; f Dèc)partement de Radiologie, Universitèc) de Montrèc)al, Montrèc)al, QC, Canada; g Laboratoire d'Imagerie Fonctionnelle • U678, Facultèc) de Mèc)decine, Pierre et Marie Curie • Pitièc) Salpèc)trière, Paris, France; h Dèc)partement de Psychologie, Unitèc) de Neuroimagerie Fonctionnelle (UNF), Universitèc) de Montrèc)al, Montrèc)al, QC, Canada

**Keywords:** Autism, fMRI, Plasticity, Primary areas, Associative areas

## Abstract

**Background:**

An enhanced plasticity is suspected to play a role in various microstructural alterations, as well as in regional cortical reallocations observed in autism. Combined with multiple indications of enhanced perceptual functioning in autism, and indications of atypical motor functioning, enhanced plasticity predicts a superior variability in functional cortical allocation, predominant in perceptual and motor regions.

**Method:**

To test this prediction, we scanned 23 autistics and 22 typical participants matched on age, FSIQ, Raven percentile scores and handedness during a visuo-motor imitation task. For each participant, the coordinates of the strongest task-related activation peak were extracted in the primary (Brodmann area 4) and supplementary (BA 6) motor cortex, the visuomotor superior parietal cortex (BA 7), and the primary (BA 17) and associative (BAs 18 + 19) visual areas. Mean signal changes for each ROI in both hemispheres, and the number of voxels composing the strongest activation cluster were individually extracted to compare intensity and size of the signal between groups. For each ROI, in each hemisphere, and for every participant, the distance from their respective group average was used as a variable of interest to determine group differences in localization variability using repeated measures ANOVAs. Between-group comparison of whole-brain activation was also performed.

**Results:**

Both groups displayed a higher mean variability in the localization of activations in the associative areas compared to the primary visual or motor areas. However, despite this shared increased variability in associative cortices, a direct between-group comparison of the individual variability in localization of the activation revealed a significantly greater variability in the autistic group than in the typical group in the left visuo-motor superior parietal cortex (BA 7) and in the left associative visual areas (BAs 18 + 19).

**Conclusion:**

Different and possibly unique strategies are used by each autistic individual. That enhanced variability in localization of activations in the autistic group is found in regions typically more variable in non-autistics raises the possibility that autism involves an enhancement and/or an alteration of typical plasticity mechanisms. The current study also highlights the necessity to verify, in fMRI studies involving autistic people, that hypoactivation at the group level does not result from each individual successfully completing a task using a unique brain allocation, even by comparison to his own group.

## Highlights

• Functional activation in associative regions are more variable in autistics than in typicals.
• Autistics showed enhanced variability in regions that are typically more variable in typicals.
• Enhanced variability follows the same rule in perceptive and motor-related regions.

## 1 Introduction

Autism is characterized by social and communication alterations, as well as by repetitive behaviors and restrictive interests, combined with a large diversity among symptomatic profiles and individual developmental trajectories (American Psychiatric Association, 2013; Newschaffer et al., 2007). The variability of autistic phenotype may result from the heterogeneity of environmental constraints and upbringing. However, mechanisms for heterogeneity may also be intrinsic to what autism is. The most obvious factor for phenotypic heterogeneity is the wide range of chromosomal regions and the several hundreds of polymorphisms that have been associated with autism (Scherer and Dawson, 2011). Whereas autism is understood as a final common pathway of these various mutations (Ben-David and Shifman, 2012), each genetic alteration may produce its own footprint on the phenotype. For instance, in the case of “syndromic autism”, autism accompanied by tuberous sclerosis will differ from that accompanied by Fragile X. Another putative source of heterogeneity may be that the common effect of these mutations (either involved in syndromic or non-syndromic autism) is an increase in synaptic plasticity, a mechanism which may increase the experience-dependent variability in brain functional allocation (Markram and Markram, 2010; Mottron et al., 2013; Chung et al., 2012; Zoghbi and Bear, 2012). However, empirical arguments in favor of enhanced plasticity in autism are mostly indirect • based on examining in animal models the effect of genetic (Kelleher and Bear, 2008; Baudouin et al., 2012) or environmental (Markram and Markram, 2010) alterations • and mostly related to *microstructural* alterations (Markram and Markram, 2010).

Enhanced *functional* plasticity should also be present at the macroscopic level, and predict a greater variability in the autism group in regional allocation of brain functions (Barnes and Finnerty, 2010). Spatial variability in functional allocation is not identically distributed on the surface of the cortex. In an fMRI resting state study in typical individuals, Mueller et al. (2013) demonstrated that functional connectivity in hetero-modal association cortices (lateral prefrontal regions, temporo-parietal junction) is substantially more variable than that in unimodal perceptual and motor cortices. Regions of this increased inter-subject variability overlap with regions displaying more variable cortical folding, as well as with regions implicated in individual cognitive differences and regions displaying the largest evolutionary expansion between monkeys and humans. Autistics should therefore present more within-group variability in terms of functional allocation in associative regions, because these regions are intrinsically more variable and less genetically constrained in humans (Brun et al., 2009). There are indications that an autistic-specific plasticity process favors these regions, as manifested by their enhanced gyrification (Wallace et al., 2013), as well as by these regions being the primary locus of structural alterations, as revealed by the latest structural meta-analysis (Nickl-Jockschat et al., 2012). At the functional level, a recent ALE meta-analysis of 26 neuroimaging experiments using visual stimuli in autistic individuals revealed a material-specific functional reallocation of visual occipitoparietal associative areas, in the form of atypical spatial distribution of neural activity, and decreased activity in some frontal areas, in autistic relative to non-autistic individuals (Samson et al., 2012).

Pierce et al. (2001) were the first to report a greater individual variability in localization of cerebral activations in autistics. Whereas hypo-activation of the fusiform gyrus was observed in autistics at the group level during a face perception task, each autistic participant had a unique functional hot spot in response to faces (ranging from the frontal lobe to the occipital lobe and fusiform gyrus), while locations in non-autistics all fell within the fusiform face area. Similar increased inter-individual spatial variability in functional activations was also found in autistic groups during a visuomotor sequence learning task (Mñ/4ller et al., 2003; Mñ/4ller et al., 2004). In these studies, the 3D distance between the group's strongest activation peak in a specific region and each individual's closest peak was used as a direct measure of individual spatial variability. The premotor (BA 6) and the superior parietal (BA 7) cortices were used as target regions. Compared to typical individuals, autistics showed greater inter-individual spatial variability and decreased activation in the right superior parietal region (BA 7) during the early learning stage, and greater variability and activation in the right premotor region (BA 6) during the late learning stage. Scherf et al. (2010) used a similar computation of the individual variability in a study involving face, object and place processing. Greater variability in localization of activations was observed within the autistic group, but only in the fusiform gyrus during face processing. Whereas these findings are consistent with our hypothesis of enhanced variability, they are post-hoc findings, and do not compare primary and associative perceptual and motor regions. This distinction is of interest because the main difference in variability reported in typical individuals involves contrasting primary and associative regions (Mueller et al., 2013; Tahmasebi et al., 2012).

The aim of the study was to use functional magnetic resonance imaging (fMRI) to determine whether there is increased inter-individual variability in the localization, intensity and size of cerebral activations within the primary and associative areas of both visual and motor modalities in autistic individuals, compared to non-autistic individuals. Between-group comparisons of whole brain activations were also performed to determine if individual variability is associated with between-group differences in task-related activity. We distinguished primary and associative areas of visual and motor modalities recruited during a visuo-motor imitation task, using anatomical ROIs. An easy visuo-motor task was chosen in order to produce a combined activation of visual and motor cortices. BA 4 (primary motor cortex), BA 6 (premotor cortex and supplementary motor area, SMA), and BA 7 (visuomotor superior parietal cortex) were selected as ROI to investigate motor functions. BA 18 (V2: secondary visual cortex) and BA 19 (associative visual cortex) were grouped together to represent the global associative areas of the visual cortex, and BA 17 (V1: primary visual cortex) composed the visual ROI.

## 2 Methods

### 2.1 Participants

The initial experimental sample comprised 26 autistic participants and 23 typically developing participants recruited from the research database of the Universitèc) de Montrèc)al Autism Center of Excellence at the Rivière-des-Prairies Hospital (Montreal, Canada). The autistic and non-autistic groups were matched on age, gender, Wechsler Full-scale and Performance IQ (WISC-III or WAIS III, Canadian norms), Raven's Progressive Matrices percentile (North American norms) (Raven, 1976) and manual preference estimated using the Edinburgh Handedness Inventory (Oldfield, 1971). Two left-handed autistics were not included in the analysis, in order to satisfy group matching in handedness. Most autistic participants were diagnosed using a multidisciplinary assessment that included a clinical evaluation based on DSM-IV criteria, the Autism Diagnostic Interview Revised (ADI-R) (Lord et al., 1994) and the Autistic Diagnostic Observation Schedule (ADOS-G modules 3•4) (Lord et al., 1989). However, some participants were characterized using expert interdisciplinary judgment only (one participant) or combined with either ADOS-G (two participants) or ADI-R (two participants). Typical participants were screened for personal or familial neurological or medical conditions known to affect brain function. Exclusion criteria were uncorrectable visual impairment, current use of psychoactive or vasoactive medications and use of drugs or alcohol exceeding 2 drinks per day. All structural scans were reviewed by a neurologist to ensure that no participant had any anatomical abnormalities. Written informed consent was obtained from all participants in accordance with the Regroupement Neuroimagerie/Quèc)bec IRB approved protocol 08-09-003. All participants received monetary compensation for their participation.

### 2.2 Stimuli and procedure

**Fig. 1:**
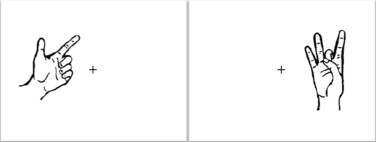
Two different sample stimuli from the visuo-motor imitation task.

The visuomotor imitation task included 15 different hand gestures drawn in black and white, each illustrated twice to represent both left and right hands. These visual stimuli were presented so that the participants saw the hands with the palm facing them and could distinguish clearly the configuration of the fingers (Fig. 1). A practice session outside the scanner ensured that the participants familiarized themselves with the different gestures, understood the task and could imitate the gestures with their hand while minimizing movement of the rest of the body. During the fMRI scanning session, participants were lying on their back in the scanner with their hands on the sides of the body, palms facing up. No visual feedback could be used during the task, as participants had to look continuously at the stimuli presented. Visual stimuli were presented using the Matlab Psychtoolbox (Brainard, 1997; Pelli, 1997; Kleiner et al., 2007), on a screen at the back of the scanner bore. The participants saw the stimuli through an individually adjusted mirror attached in front of their eyes on the head coil. Vision correction with fMRI compatible lenses for participants with myopia or hyperopia was applied in concordance with their optometrist's prescription. A total of 96 hand gestures were presented during the session, in 16 blocks of 6 trials. The session started with a 10 second fixation cross. Then, each of the 16 blocks included a 2.5 second instruction slide indicating the hand to be used to imitate the hand gesture presented (left hand or right hand condition) for the following 6 trials. The stimuli were presented pseudo-randomly (3 s/stimulus) in the same visual field as the hand to be used to imitate. A fixation cross (9.5 second duration) ended each block and served as the baseline. The total duration of the session was 490 s.

### 2.3 Image acquisition

Images were acquired on a Siemens Tim Trio 3T scanner with a 32 channel phased-array head coil at the “Unitèc) de Neuroimagerie Fonctionnelle” (University of Montreal). The scanning session included anatomical T1-weighted structural brain images using an ME-MPRAGE 4-Echo sequence (176 slices, 1 mm^3^ voxels, TR = 2530 ms, TE = 1.64/3.5/5.36/7.22 ms, flip angle = 7°). Acquisition of functional data used an echo planar imaging (EPI) pulse sequence (150 acquisitions, TR = 3330 ms, 60 slices, matrix size 80 í 80, voxel size 2.5 í 2.5 í 2 mm^3^, slice thickness: 2 mm with a 0.5 mm gap, TE = 30 ms, flip angle = 90°). Gradient echo phase and magnitude field maps were then acquired (60 slices, matrix size = 80 í 80, voxel size 2.5 í 2.5 í 2.0 mm^3^, slice thickness = 2 mm with a 0.5 mm gap, TR = 488 ms, TE short = 4.92 ms, TE long = 7.38 ms, flip angle = 60°) to correct image distortions and improve co-registration accuracy using the field map toolbox in SPM.

### 2.4 Image analysis

SPM8, MRICRON and SPSS were respectively used for image preprocessing, visualization and statistical analysis.

#### 2.4.1 Preprocessing

Image preprocessing steps started with a two-pass realignment involving initial registration of all images to the first image of the time series within the run, followed by registration of the images to the mean of the images computed after a first realignment, and then followed by resampling using 4th degree b-spline interpolation. Slice time correction was applied using interpolation between time points at each voxel taking the last slice of the EPI volume as reference. Images were then spatially transformed and spatially normalized into the ICBM152 MNI space. Normalized images were finally smoothed using 3D Gaussian filtering kernel of 8 mm FWHM.

#### 2.4.2 Statistical modeling

After inspection of functional activations (at uncorrected *p* < .001), participants were excluded if they presented an aberrant pattern of activation with no activation in the visual and motor-related areas (1 per group). The final sample included 23 autistic and 22 typical participants (Table 1). Head motion parameters during the functional scanning session were inspected and did not exceed 1.5 mm of translation and 0.05 degree of rotation for any of the participants. Independent-sample *t*-tests were performed on the translation and rotation parameters. The groups did not differ in the magnitude of maximal translation (*t*(43) = .624, *p* = .536) or rotation (*t*(43) = ∧.724, *p* = .473).

To allow longitudinal magnetization equilibration, the first two volumes of the session were discarded. Model specification of the first level analysis included a design matrix for each of the two active conditions (left/right hand) and the baseline condition (fixation cross) corresponding to the timing described above in the procedure in Section 2.2. Six head motion estimates were included in the model as covariates of no interest. A high-pass temporal filter with a cutoff of 128 s was also used to remove low-frequency noise. A GLM model was used for statistical analysis. The hemodynamic response was modeled using the canonical hemodynamic function implemented as boxcar basis functions in SPM8. In the first-level analysis, the following contrasts were computed: left and right hand respectively vs. the fixation cross baseline. To allow inference at the population level, the mixed effect model included a second-level analysis where the first-level contrasts were entered in a random-effect model with three factors: Subject (55 levels), Group (2 levels), which was assumed to have unequal variance, and Condition (2 levels). The mixed effect model covers the first-level analysis (accounting for within-subject variability) followed by second level analysis (accounting for between-subject variability). The critical threshold was *t* = 5.38, *p* < .05, FWE with an extent threshold of *k* = 20 voxels.

### 2.5 Computing parameters of individual variability

*Individual variability* corresponds to the magnitude of the within-group variability, and is measured through three different parameters based on the strongest activation peak of task-related activity: its *localization*, its *mean signal change* between task and baseline and its *size*. In order to compute individual variability for these three parameters, regions of interest (ROIs), which were defined from a Brodmann area (BA) atlas using the WFU Pickatlas SPM Toolbox (Maldjian et al., 2003; Maldjian et al., 2004), were used to measure the individual activations in the visual and motor regions involved in the task. Using these ROIs defined from a template rather than from each individual is justified for this study because the size of individually defined ROIs depends on the statistical significance of the functional responses, which were a function of the variability and the response amplitude (Dinstein et al., 2010). As the goal of this study is to investigate individual variability in autistic compared to typical individuals, the use of anatomically defined ROI masks gave us a legitimate comparison point. Computations of the three parameters determining individual variability were all based on the strongest activation peak for each ROI, within each hemisphere. Separate repeated measures ANOVAs were performed for the visual and motor modalities with Region (primary, associative), Side (left, right) and Group as factors. Significant main effects and interactions were then further investigated using two-tailed independent-samples *t*-tests. Since this measure of variability is associated with standard deviation of the parameters computed, Levene's tests (homogeneity of variance) were applied.

#### 2.5.1 Spatial localization

Coordinates of the strongest activation peak were extracted from the functional images with an uncorrected threshold (*p* < .001) for each participant, ROI and hemisphere. Using a lower uncorrected threshold is justified given our goal, as it does not affect the localization of the activation, only its intensity and its size. Based on the method used by Mñ/4ller et al. (2003), distances in three-dimensional stereotactic space were computed between the group mean maxima and the individual maxima. For example, if the strongest activation in the right BA 17 for one participant was located at [20, ∧84, 10] and occurred at [18, ∧93, ∧4] for the group mean, the distance was $\sqrt{2^{2}+9^{2}+14^{2}=16.76}$ mm. The variable obtained was thus the geometric distance from the group mean activation, and was therefore used to measure intra-group variability in localization of activations, or spatial variability.

#### 2.5.2 Mean signal change

To compare the intensity of the signal between groups, mean signal changes of activated voxels of each individual for each ROI and hemisphere were extracted from the strongest activation peak (Chung et al., 2007). Only those which reached the more conservative threshold of *p* < .05 FWE corrected were included in the analyses. An average of 2 measures was excluded in each group and ROI, except for the BA 7 ROI; about 7 measures had to be excluded in each group.

#### 2.5.3 Size of activation

The size of activation was determined by computing the number of voxels that reached the conservative threshold of *p* < .05 FWE corrected within the ROI.

### 2.6 Group analysis: whole brain task-related activity

In order to disentangle region-specific individual variability from group differences in whole brain activation, the latter was computed through a repeated measures ANOVA with Subject, Group (typical or autistic) and Condition (left hand or right hand) as factors.

### 2.7 Voxel-based morphometry

#### 2.7.1 Image preprocessing

A voxel-based morphometry (VBM) analysis was conducted using the SPM8 VBM-DARTEL procedure (Ashburner, 2010) to see whether the group differences observed in terms of functional variability could be explained by an anatomical difference in the same gray matter regions. First the T1 images were visually inspected for artifacts and 3 subjects were rejected at this level. The images were segmented into gray matter (GM), white matter (WM) and cerebrospinal fluid (CSF) using the *New Segment* tool. The resulting gray matter images for each subject were then used in the DARTEL (create templates) procedure. The resulting template files of each subject were smoothed, spatially normalized and Jacobian scaled to MNI space. A 10 mm FWHM Gaussian kernel was used.

#### 2.7.2 Statistical analysis

A *t*-test was performed to investigate whole-brain group differences in gray matter correcting for total intra-cranial volume (GM + WM + CSF) using global normalization.

## 3 Results

### 3.1 Individual variability differences

#### 3.1.1 Spatial localization

**Fig. 2:**
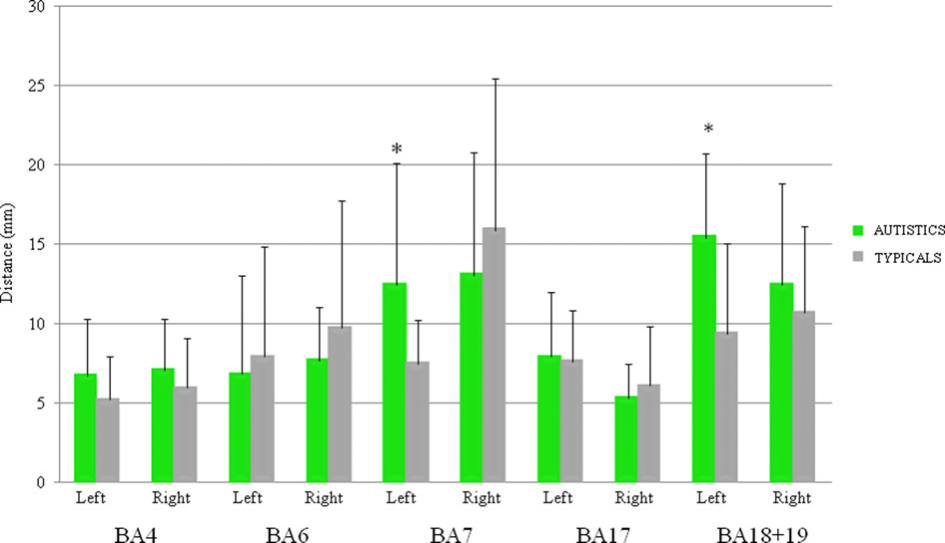
Mean distances in millimeters from the group mean activation peak in the motor and visual ROIs in autistic and typical groups during the visuo-motor imitation task. Differences between groups reaching significance (*p* < .05) are indicated by an asterisk (*).

(Fig. 2) Mean distances and their standard deviation for each group, ROI and hemisphere are presented in Table 2.

##### 3.1.1.1 Visual areas

**Fig. 3:**
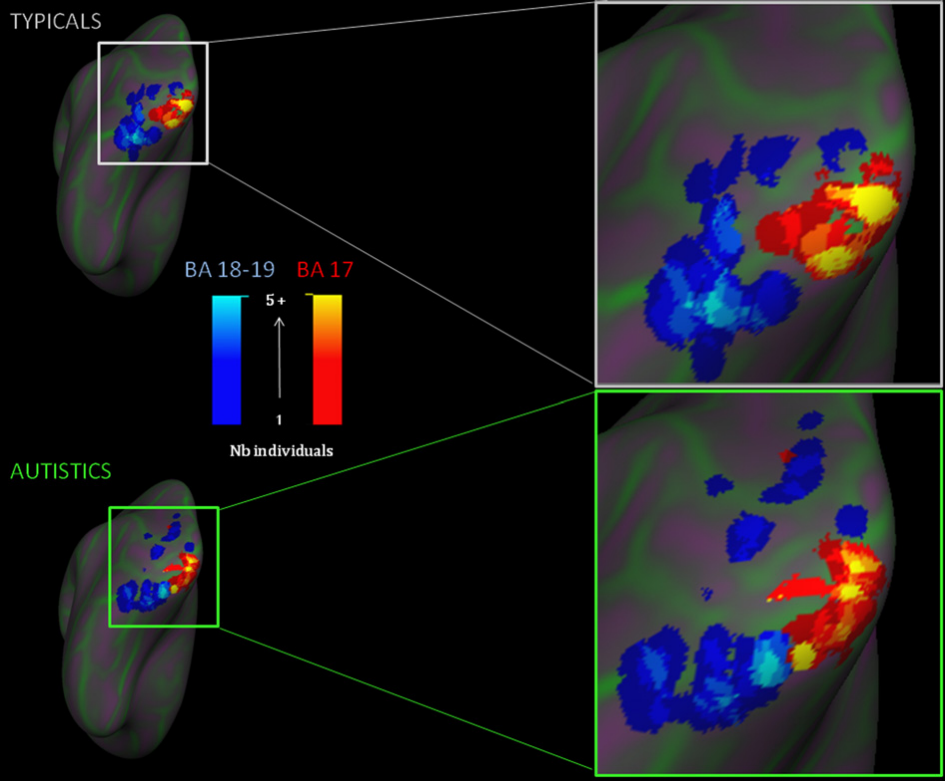
Localization of all individual peaks of activation in the left visual ROIs. Each peak is represented as a 1 cm diameter sphere projected on the cortical surface using Freesurfer. The color scale represents the overlap of individual peaks, darker being 1 individual and brighter 5 and more. The primary area (BA 17) is in red and the associative area (BAs 18•19) is in blue. The autism and typical groups are displayed separately.

The repeated measures ANOVA revealed significant Region (*F*(1,39) = 50.79, *p*< .001) and Side (*F*(1,39) = 5.15, *p*= .029) main effects, as well as a Region í Group interaction (*F*(1,39) = 6.38, *p*= .016). In both groups the variability was more important in the left hemisphere. Independent *t*-tests showed that the variability of individual distances from the group mean activation peaks was greater in the autistic group than in the typical group in left BAs 18 + 19 (associative visual regions: *t*(43) = 3.67, *p*= .001) but not in left BA 17 (*t*(41) = .257, *p*= .799). This group difference was not present for the right hemisphere (right BAs 18 + 19: *t*(42) = .979, *p*= .333, right BA 17: *t*(40) = ∧.821, *p*= .417) (see Fig. 3). Levene's tests did not reach significance.

##### 3.1.1.2 Motor-related areas

**Fig. 4:**
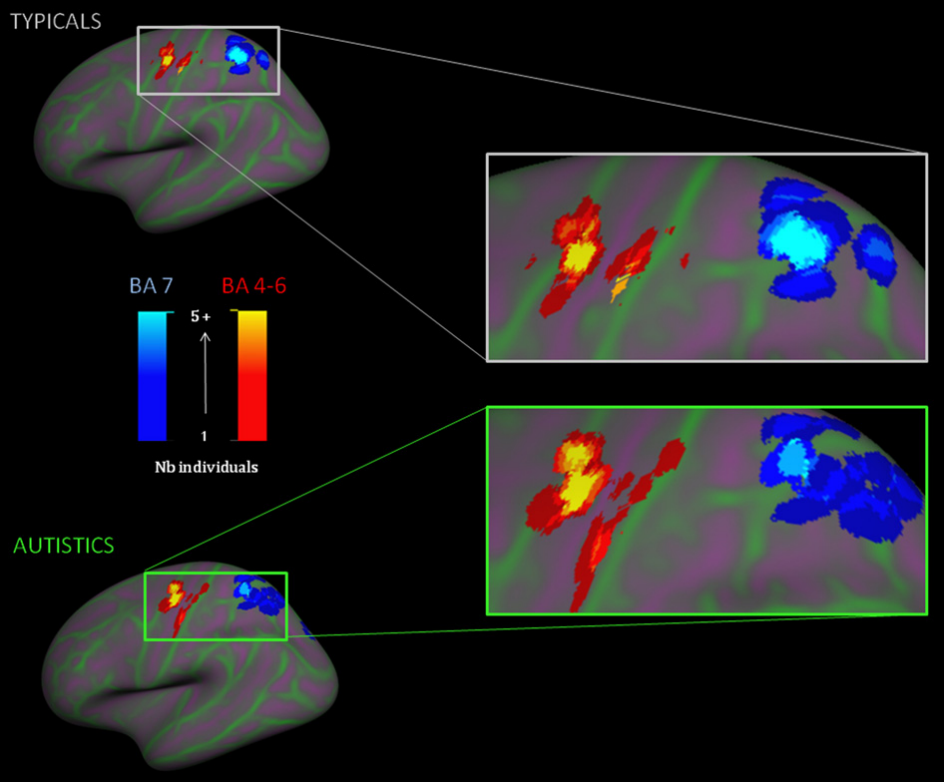
Localization of all individual peaks of activation in the left motor ROIs. Each peak is represented as a 1 cm diameter sphere projected on the cortical surface using Freesurfer. The color scale represents the overlap of individual peaks, darker being 1 individual and brighter 5 and more. The primary area (BAs 4•6) is in red and the associative area (BA 7) is in blue. The autistic and typical groups are displayed separately.

The repeated measures ANOVA revealed significant Region (*F*(1,35) = 26.29, *p*< .001) and Side (*F*(1,35) = 11.72, *p*= .002) main effects, a Side í Group interaction (*F*(1,35) = 6.06, *p*= .019) as well as a three-way Region í Side í Group interaction (*F*(1,35) = 6.00, *p*= .019). The independent *t*-tests revealed that autistics exhibited greater variability than typicals in BA 7 only and on the left side only (*t*(37) = 2.67, *p*= .014) the latter being associated with a significant Levene's test (*p*= .032) as well. The independent *t*-tests and Levene's tests did not reach significance (*p*> .05) for the between-group differences in the primary motor ROIs (BA 4, BA 6) (see Fig. 4).

#### 3.1.2 Mean signal change

**Fig. 5:**
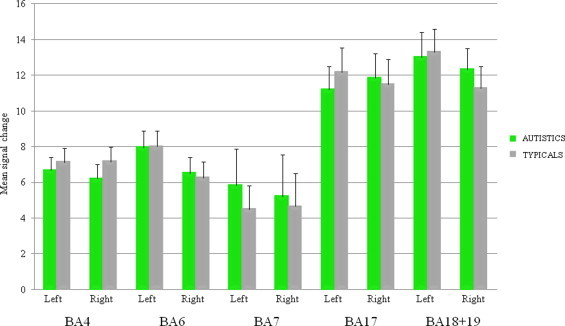
Mean signal change in motor and visual ROIs in autistic and typical groups during the visuo-motor imitation task. No difference between groups was observed.

No significant between-group difference was observed in mean signal change (*p*> .05) (Fig. 5). Levene's tests were not significant, except for the left BA 4 (*p*= .039).

#### 3.1.3 Size of activation

**Fig. 6:**
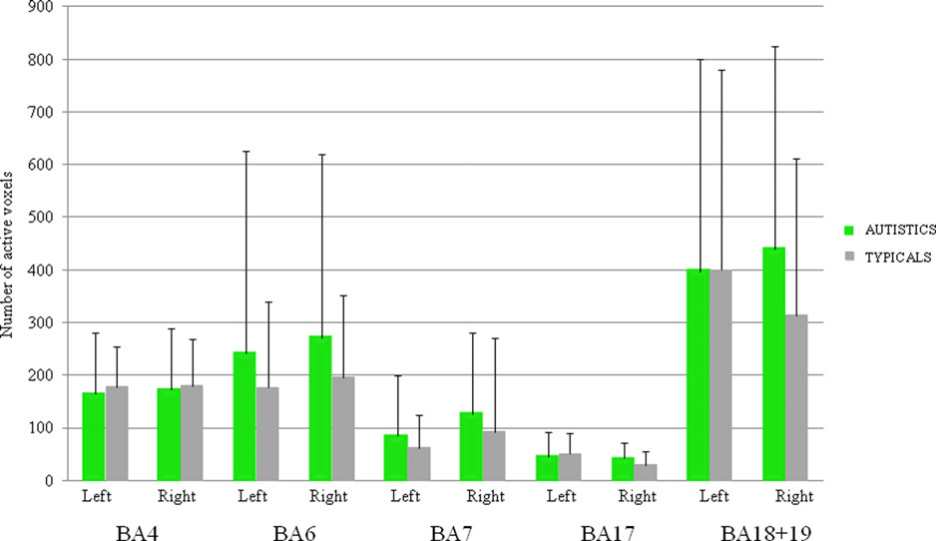
Mean size of activation in motor and visual ROIs in autistic and typical groups during the visuo-motor imitation task. No difference between groups was observed.

(Fig. 6) No significant between-group difference was observed in the size of activation (*p*> .05). Levene's tests were not significant.

### 3.2 Group analysis: whole brain task-related activity

#### 3.2.1 Within-group contrasts

In each group, every ROI showed functional engagement during the task. Both groups showed the same pattern of activation in the middle occipital gyrus, inferior semi-lunar lobule and middle frontal gyrus. However, unlike the autistic group, the typical group recruited frontal regions, including superior, inferior and superior frontal gyrus. The autistic group showed activations in the middle occipital gyrus, inferior semi-lunar lobule, nodule and caudate, regions that the typical group did not significantly recruit. Results are presented in Table 3.

#### 3.2.2 Between-group contrasts

For the *left hand* condition, between-group contrasts revealed greater activation for the autistic group compared to the typical group in the left lingual gyrus (BA 19), while the typical group showed greater activation in the middle occipital gyrus (BA 19) and the middle temporal gyrus (BA 39) compared to autistics (*p*< .05, FWE). For the *right hand* condition, typical individuals showed the same pattern of increased activity in the middle occipital gyrus (BA 19) and the middle temporal gyrus (BA 39) (*p* < .05, FWE). No region was significantly more active in the autistic group than in the typical group for the *right hand* condition. When *left and right hand* conditions were combined, typical individuals still showed greater activation in the middle occipital (BA 19) and middle temporal gyrus (BA 39) bilaterally, as well as in the left inferior occipital gyrus (BA 18) (*p*< .05, FWE). These results are consistent with those obtained by Tanaka and Inui (2002) and Mñ/4hlau et al. (2005) in similar imitation tasks. The autistic group showed greater activity than the typical group in a number of regions involved in visual and motor processing: bilateral middle occipital gyrus (BA 19 and BA 18) in a more superior portion than the typical group's active region, left cuneus (BA 18), lingual gyrus (BA 18 + BA 19) and precuneus (BA 31) bilaterally, right medial frontal gyrus (BA 6) and superior frontal gyrus (BA 6) and bilateral superior parietal lobule (BA 7). Results are shown in Table 4.

### 3.3 Voxel-based morphometry

No significant difference was observed between groups in terms of regional gray matter increases or decreases (with a threshold of FWE *p*< .05).

## 4 Discussion

The aim of this study was to test the prediction that, given enhanced plasticity in autism, this group should display increased spatial variability of activations in motor and visual associative areas. We also investigated between-group differences in the magnitude of activation in these regions, which may interact with this variability. Consistent with our prediction, inter-individual variability in localization of activation was greater in the autistic, as compared to the typical group, in the left associative visual areas (BAs 18 + 19) and in the superior parietal cortex (BA 7). These results are not likely to be explained by anatomical gray matter variability, since the VBM analysis did not reveal any group differences. No group differences in terms of variability of intensity and size of the activations were observed.

### 4.1 Validity and sensitivity of individual variability measurements

Individual variability in *localization* was measured using the Euclidean distance between the stereotactic coordinates of an individual's strongest activation peaks and that of the group mean. Compared to a surface-based analysis, drawing the most direct line between two activation peaks underestimates their actual distance, as it neglects the gray matter curvilinear morphology. However, despite its limits in precision, the use of Euclidean distance for this purpose has been well documented and validated. In addition, the effective slice thickness limits the resolution of spatial distances to 2 mm but this bias is shared by the two populations under study, and therefore should not mask group differences. The mean distances (between 4 and 23 mm) computed per regions and hemispheres were largely above the spatial resolution used in this study. Our technique can therefore be considered as a satisfying measure of localization variability.

The absence of behavioral measures prevents us from disentangling group differences in topographical brain activity from those associated with performance. However, whether or not the groups differed in terms of accuracy in the imitation task should not affect our results as we were interested in within-group individual variability. Since all participants trained successfully at the task before scanning, and since this elementary task is performed at ceiling level in adults of average measured intelligence (Salowitz et al., 2013; Williams et al., 2004), the possible role of within-group difference in performance variability in our findings should be minimal.

Another limitation of this study is that individual variability was assessed using a single measure per subject. Mueller et al. (2013) used several measures taken 6 months apart and subtracted intra-subject variability from overall variability to obtain residual inter-subject variability. Our single-measure procedure may therefore overestimate inter-subject variability. However, it could not overestimate group differences in this regard, as the two groups shared this bias.

### 4.2 Individual variability in localization of visual and motor activations

#### 4.2.1 Primary and associative visual areas

No difference related to localization of activation was observed in either left of right primary visual cortices (BA 17). A similar magnitude of activation in primary visual areas (BA 17) in both groups is concordant with Hadjikani et al.'s (2004) findings that the early sensory visual areas are typically organized in autistic adults. Although a greater variability could be influenced by a greater task-specific cognitive demand in autistic participants, greater variability parameters in the associative than in the primary areas was shared by both groups. The fact that mean variability in the localization of activations was higher in the associative than in the primary visual areas for both groups is consistent with associative areas being more variable than primary perceptual ones (Mueller et al., 2013). It is also in line with the increase in variability of localization paralleling the hierarchy of levels of processing (Tahmasebi et al., 2012).

The main difference between groups in terms of variability resided in an even greater variability in localization of activation in autistic than in typical individuals in the left associative visual areas (BAs 18 + 19). The autistic group displayed greater bilateral activation in the middle occipital gyrus (BA 18 + BA 19), lingual gyrus (BA 18 + BA 19) and precuneus (BA 31), as well as in the left cuneus (BA 18). A greater variability in associative visual regions in the autistic group is consistent with the greater implication of the associative visual areas during tasks involving visual stimuli in autism (Samson et al., 2012), and particularly BA 18 (Soulières et al., 2009).

#### 4.2.2 Primary and associative motor-related areas

Following a pattern similar to that of visual regions, mean distances between the individual activation peak and the group mean activation peak of each group drastically increased between BA 4 and BA 7. This trend is more pronounced in autistics, who showed greater spatial variability of activations in the left hemisphere in BA 7, an associative visuomotor region, while no difference was observed in the primary motor area, BA 4, and premotor cortex/SMA, BA 6. BA 7 is localized in the superior parietal cortex and is involved in the integration of visual and motor information. This region receives afferences from the visual areas and sends information to the premotor areas (BA 6). The second level analysis revealed that the autistic group showed greater activation than the typical group in the right medial and superior frontal gyrus (BA 6) and in the bilateral superior parietal lobule (BA 7). Increased activation in the superior parietal cortex was also reported in autism during a visuo-spatial task (Damarla et al., 2010) and was associated with a greater functional importance of the visuo-spatial processing in autistics compared to typicals. Our results are consistent with the behavioral literature of motor skills in autism, showing an atypical role of sensory•perceptual input/feedback in autistics when executing a motor task (Izawa et al., 2012; Linkenauger et al., 2012; Gowen and Hamilton, 2013).

#### 4.2.3 Effect of lateralization

The three series of group differences reported here are all limited to the left hemisphere. This may at least partially result from autistics displaying an atypical reduction of lateralization in functions which are usually lateralized in typical individuals. In the autism literature, most differences in lateralized functions independent of handedness are in the form of an absence of asymmetry in language-related regions (Herbert et al., 2002; De Fossèc) et al., 2004; Rojas et al., 2002, 2005) and face processing areas (Conturo et al., 2008; Kleinhans et al., 2008, 2010). However, since the visual and motor functions under study here are not known to be lateralized, and the variability under study in this paper is not directly associated with superior activation, we do not know if this explanation can be applied to the current set of findings.

### 4.3 Mechanisms of topographical variability

In the context of atypical microstructural plasticity and multiple de-novo mutations of genes involved in the construction of local neural networks in autism (Kelleher and Bear, 2008), an increased variability of cortical functional allocation may be attributed to the alteration of the neurobiological and experience-dependent plasticity mechanism responsible for this variability in typical individuals (Mottron et al., 2013; Mueller et al., 2013). Regions of enhanced variability in autistics are, at least partially (for visual tasks), overlapping with regions also displaying an enhanced activity, and are functionally associated with peaks of ability (Soulières et al., 2009). This suggests that this alteration of dominant functional allocation is related to one of the most specific aspects of autistic cognition. Moreover, considering that perceptual peaks of performance are not found to the same extent in autistic people with and without speech onset delay (Bonnel et al., 2010; Barbeau et al., 2013), this variability may also contribute to the difference between the autism and Asperger subgroups, based on contrasted speech acquisition and perceptual performance (Mottron et al., 2013).

### 4.4 Methodological consequences of variability

Finally, the fact that individual variability of the functional allocation of brain resources interferes with between-group differences in activations has important heuristic consequences for future fMRI studies of autistic people. Our results are consistent with those of Mñ/4ller et al. (2001) and Pierce et al. (2001) in that the autism group is characterized by spatial inconsistencies in the activations across subjects, as can be observed in Figs. 3 and 4 of the present study; sites of activations specific to one individual are more numerous and distant in the autism group than in the typical group. Autistic•control fMRI group differences are usually interpreted as evidence of functional deficits at the group level (Gernsbacher, 2007). The current study suggests, rather, that hypoactivation at the group level may result from each individual successfully completing a task using a unique brain allocation, even by comparison to his own group. This is confirmed by an ALE meta-analysis (Samson et al., 2012) demonstrating that heterotopic activation can coexist with typical group performance. This highlights the necessity to assume that, for fMRI second level analysis, autistic samples have unequal variance, particularly when the task involves motor and perceptive associative regions. We therefore encourage an investigation of individual variability, including its measurement using distance computation between individual activations and their group mean activations, or using surface-based analysis.

## 5 Conclusion

Preliminary investigations of autistic topographical variability in task-related brain activation (Pierce et al., 2001; Scherf et al., 2010) reveal greater and more heterogeneous implication of the associative visual areas. Our study adds a new element to the interpretation of this variability: in the visual and motor-related domain, autistics display an increased functional variability in the regions where typical individuals also show enhanced topographical variability relative to other regions, raising the possibility that autism involves an enhancement and/or an alteration of typical plasticity mechanisms.

## Figures and Tables

**Table 1:** Participant characteristics.

	Typicals	Autistics	*p* value
**Sample size (gender)**	22 (3F, 19 M)	23 (3F, 20 M)	
**Age (years)**			
Mean (SD)	22.6 (5.56)	19.8 (4.72)	.125
Range	15•35	14•30	
**Full-scale IQ**			
Mean (SD)	107.3 (12.51)	100.3 (10.48)	.056
Range	87•127	86•118	
**Performance IQ**			
Mean (SD)	104.7 (13.14)	105.1 (11.74)	.709
Range	82•122	92•127	
**Verbal IQ**			
Mean (SD)	108.7 (11.81)	99.8 (14.87)	.017
Range	91•127	67•119	
**Raven percentile**			
Mean (SD)	68.1 (25.41)	75.5 (15.99)	.268
Range	25•96.5	50•100	
**Handedness**			
Mean (SD)	+74.05 (38.57)	+62.35 (59.06)	.460
Range	∧87.5 to +100	∧80 to +100	
**ADOS score**			
Mean (cut-off)			
Communicative	∧	4.95 (3)	
Social	∧	9.57 (6)	
Social + communicative	∧	14.52 (10)	
**ADI score**			
Mean (cut-off)			
Social	∧	21.48 (10)	
Communicative	∧	16.86 (8)	
Behavior	∧	6.14 (3)	

Note. Groups were matched on gender, age, full scale IQ, performance IQ, Raven Progressive Matrices percentile scores and manual preference, which is reported as the Edinburgh score (from ∧100 completely left handed to +100 completely right handed). ADI: Autism Diagnostic Interview, ADOS: Autism Diagnostic Observation Schedule. Group differences were assessed using independent *t*-tests.

**Table 2:** Mean distances from the individual activation peak to the group mean activation peak, and standard deviation for each group, ROI and hemisphere.

	Typicals	Autistics
Region and hemisphere	Mean (SD)	Mean (SD)
**Visual**		
BA 17 left	7.63 (3.22)	7.92 (4.08)
BA 17 right	6.08 (3.72)	5.32 (2.12)
BAs 18 + 19 left*	9.37 (5.68)	15.41 (5.35)
BAs 18 + 19 right	10.72 (5.39)	12.45 (6.40)
**Motor**		
BA 4 left	5.21 (3.69)	6.74 (3.56)
BA 4 right	5.96 (3.09)	7.09 (3.22)
BA 6 left	7.94 (6.89)	6.85 (6.13)
BA 6 right	9.75 (7.98)	7.69 (3.37)
BA 7 left*	7.49 (2.76)	12.44 (7.64)
BA 7 right	15.87 (9.61)	13.02 (7.74)

Note. Distances are in millimeters. Significant group differences (*p* < .05) are indicated by an asterisk (*).

**Table 3:** Activity associated with the visuo-motor imitation task in each group.

		Left						Right				
Region label	BA	*x*	*y*	*z*	*t*	*d*		*x*	*y*	*z*	*t*	*d*
**TYPICALS**												
**Both conditions**												
*Occipital*												
Middle occipital gyrus	18	∧36	∧86	∧8	52.09	15.92		28	∧86	∧8	46.93	14.35
		∧28	∧86	4	47.20	14.42						
*Posterior*												
Inferior semi-lunar lobule		∧26	∧62	∧52	13.30	4.07		30	∧60	∧52	10.33	3.16
		∧20	∧70	∧50	11.41	3.49		20	∧72	∧48	10.11	3.09
*Frontal*												
Superior frontal gyrus	9	∧42	42	32	12.39	3.78						
		∧34	54	32	6.72	2.05						
Inferior frontal gyrus	46							52	40	12	9.48	2.89
Middle frontal gyrus	10							42	40	8	6.49	1.98
Superior frontal gyrus	9							38	48	34	9.44	2.88
												
**AUTISTICS**												
**Both conditions**												
*Occipital*												
Inferior occipital gyrus	18	∧34	∧84	∧10	47.67	14.57						
Middle occipital gyrus	18	∧30	∧88	∧2	40.21	12.29		28	∧86	∧8	45.91	14.03
*Posterior*												
Inferior semi-lunar lobule		∧18	∧62	∧52	11.17	3.41		22	∧64	∧52	9.52	2.91
		∧28	∧58	∧52	9.96	3.04		14	∧76	∧46	8.67	2.65
		∧14	∧72	∧48	9.13	2.79						
*Frontal*												
Middle frontal gyrus	9							42	46	32	10.17	3.11
								36	38	28	6.95	2.14
*Anterior lobe*												
Nodule								2	∧56	∧34	6.66	2.04
*Sub-lobar*												
Caudate		∧18	28	∧4	6.89	2.11						

Note: Specific activations for each group in both conditions (left hand + right hand). The coordinates are in MNI space. BA refers to Brodmann area and *d* represents Cohen's effect size. The critical threshold was *t*= 5.38, *p*< .05, FWE. Extent threshold: *k*= 20 voxels.

**Table 4:** Between-group differences in the visuo-motor imitation task.

		Left						Right				
Region label	BA	*x*	*y*	*z*	*t*	*d*		*x*	*y*	*z*	*t*	*d*
**TYPICALS > AUTISTICS**												
**Both conditions**												
*Occipital*												
Inferior occipital gyrus	18	∧40	∧86	∧4	8.29	2.53						
Middle occipital gyrus	19	∧30	∧84	4	8.40	2.56		44	∧74	0	8.48	2.59
*Temporal*												
Middle temporal gyrus	39	∧44	∧76	14	6.27	2.46		54	∧72	10	7.41	2.26
								58	∧62	4	6.66	2.03
**Left hand condition**												
*Occipital*												
Middle occipital gyrus	19							44	∧74	0	6.68	2.59
*Temporal*												
Middle temporal gyrus	39							54	∧72	10	6.20	2.26
**Right hand condition**												
*Occipital*												
Middle occipital gyrus	19	∧30	∧84	4	6.32	2.56						
		∧38	∧86	6	6.07	2.34						
*Temporal*												
Middle temporal gyrus	39	∧46	∧76	14	6.70	2.39						

**AUTISTICS > TYPICALS**												
**Both conditions**												
*Occipital*												
Middle occipital gyrus	19	∧32	∧84	18	8.55	2.61						
	18							38	∧92	10	6.33	1.93
								14	∧94	14	6.76	2.06
Precuneus	31	∧20	∧76	20	6.60	2.01		26	∧78	22	7.67	2.34
Cuneus	18	∧2	∧86	18	6.58	2.01						
Lingual gyrus	19							30	∧88	24	6.17	1.88
	18	∧20	∧70	∧6	6.72	2.05		18	∧72	∧8	6.61	2.02
								20	∧78	∧16	6.25	1.91
*Parietal*												
Superior parietal lobule	7	∧22	∧74	50	7.66	2.38		28	∧70	52	6.96	2.12
Postcentral gyrus	2	∧32	∧28	38	6.21	1.89						
*Sublobar*												
Caudate		∧18	28	∧4	6.61	2.02						
*Frontal*												
Superior frontal gyrus	8							26	34	52	6.37	1.94
Medial frontal gyrus	6							6	∧20	70	6.16	1.88
**Left hand condition**												
*Occipital*												
Lingual gyrus	19	∧24	∧68	∧8	6.00	1.89						
**Right hand condition**												
No significant loci												

Note. Activity associated with group differences in both conditions (left hand + right hand), left hand condition and right hand condition. The coordinates are in MNI space. BA refers to Brodmann area and *d* represents Cohen's effect size. The critical threshold was *t*= 5.38, *p*< .05, FWE. Extent threshold: *k*= 20 voxels.
